# Fibromyalgia in Health Care Worker During COVID-19 Outbreak in Saudi Arabia

**DOI:** 10.3389/fpubh.2021.693159

**Published:** 2021-09-10

**Authors:** Fahidah AlEnzi, Sara Alhamal, Maryam Alramadhan, Ahmed Altaroti, Intisar Siddiqui, Ghada Aljanobi

**Affiliations:** ^1^Clinical Sciences Department, College of Medicine, Princess Nourah Bint Abdulrahman University, Riyadh, Saudi Arabia; ^2^Department of Internal Medicine, Rheumatology Section, Qatif Central Hospital, Al Qatif, Saudi Arabia; ^3^Department of Dental Education, College of Dentistry, Imam Abdulrahman Bin Faisal University, Dammam, Saudi Arabia

**Keywords:** COVID-19, fibromyalgia, health workers, Saudi Arabia, LFESSQ tool, FiRST

## Abstract

**Background:** In the face of the contemporary COVID-19 pandemic, health service providers have emerged as the most at-risk individuals who are likely to contract the severe acute respiratory syndrome coronavirus 2 (SARS-CoV-2).

**Aim:** To measure the prevalence of fibromyalgia (FM) during COVID outbreak among health workers in Saudi Arabia using FiRST and LFESSQ tool.

**Methods:** The study employed a cross-sectional methodology to explore the prevalence of Fibromyalgia among health workers at different health care settings in Saudi Arabia. The assessment of the prevalence of fibromyalgia among health worker was determined by using the Fibromyalgia Rapid Screening Tool (FiRST) and London Fibromyalgia Epidemiological Study Screening Questionnaire (LFESSQ) questionnaires. Descriptive statistics were used to summarize the data.

**Results:** The sample size included 992 participants. The prevalence of fibromyalgia using FiRST and LFESSQ was 12.6 and 19.8%, respectively. In this study, the prevalence of fibromyalgia was higher in females when compared to males. Most of the respondents have Vitamin D deficiency. The relationship of fibromyalgia was significantly associated with the participants who worked during an outbreak, who covered COVID-19 inpatient, covered in-hospital on call and in area quarantine.

**Conclusion:** The study's findings demonstrate that the prevalence of Fibromyalgia among health service providers during the current COVID-19 pandemic is considerably higher and that there are potential interventions that may be employed to mitigate the prevalence of the infection during the COVID-19 crisis.

## Introduction

A key concern for most nations across the globe currently is the increasing rates of SARS-CoV-2 infections (1). On 30 January 2020, COVID-19 was declared a global public health concern. By 8 March 2020, the World Health Organization (2) identified that the crisis was now a pandemic. SARS-Zoonotic Covid-2 origin is unknown, however it is genomic sequence was shown to be closely related to two bat-born SARS-like coronaviruses (3). With a population of about 34 million individuals, Saudi Arabia is identified as the second-largest country in the Arab world. Thirty-seven percent of the country's population comprises of individuals that are not from the native Saudi community. Most of the population is in the middle age group of 15–64 years, while 32.4 and 2.8% are in the 0–14 and >65 age groups, respectively (4). By December 2020, the country had recorded over 359,415 confirmed cases of COVID-19 infections and 6,012 mortalities related to the infection. A significant proportion of the COVID-19 cases in the country have been linked to the thousands of returning travelers who unconsciously contracted and transmitted the disease to the local population and particularly their immediate contacts (5). COVID-19 disease has resulted in millions of morbidities and deaths over the world. Currently, there are no effective treatment available for COVID-19 however, numerous therapies have been tested to contain COVID-19 (6).

Across the globe, the COVID-19 pandemic is placing a massive strain on the healthcare system that was stretched even before the pandemic. In the COVID-19 outbreak, the healthcare workers are at the frontline, facing hazards that include psychological violence, occupational burnout, psychological distress, fatigue, long working hours and pathogen exposure (2). As a result, these factors can have a negative effect on health care workers' mental and physical health, which can manifest as sleep disruptions, faulty eating patterns in response to stress, unbalanced diet, weight gain and anxiety (7, 8). According to a study carried by Nguyen et al. were conducted in UK and USA, they found that health care workers were more likely to have a positive COVID-19 result (9). Overall, the health care workers are on the frontline, and they are in close contact with infected patient as well as infected doctors and nurses will lead to more psychological stress to their colleagues and to the health system (6), and also the development of fibromyalgia (10). Generally, fibromyalgia (FM) increases during stressful situations and after traumatic exposure. FM is considered a long-lasting condition which is accompanied by key indicators of cognitive dysfunction, sleep disturbance, fatigue, stiffness, and widespread pain (11, 12). One of the most prominent disorders seen by primary care physician is FM. FM patients also have a bewildering variety of symptoms and a lack of reliable outcomes, which may frustrate their health care providers' diagnostic efforts (12). FM is not an uncommon condition in epidemiological terms, and its approximate prevalence differs in relation to the service group and methods. The proportion of patients visiting general practitioners is estimated to be 2–6%, 5–8% in admitted service users, and 14–20% in rheumatology medical enquiries (13).

Several self-questionnaires including FM impact questionnaire (FIQ), fibromyalgia survey questionnaire (FSQ), fibromyalgia impact questionnaire revised (FIQR), etc. these questionnaires have been developed and validated for FM (14–16). The Fibromyalgia Rapid Screening Tool (FiRST), developed by the French Society of Rheumatology and validated by Perrot et al., is currently the fastest and best performing tool (17). It is used to detect FM in <3 min with a specificity and sensitivity of 85.7 and 90.5%, respectively. The London Fibromyalgia Epidemiological Study Screening Questionnaire (LFESSQ) was another tool developed and validated for fibromyalgia screening by White et al. (18). It has two components that includes pain and fatigue, and has been tested in the general population, unlike the FiRST. Although the impact of COVID-19 on the general population has been reported in various studies (19–21), however, there are none evaluated the prevalence of FM during COVID outbreak among health workers specifically using different screening tools and factors associated with its development. Therefore, the current study aims to examine the prevalence of FM during COVID-19 outbreak among health workers in Saudi Arabia using FiRST and LFESSQ tool.

## Materials and Methods

This is a cross-sectional study among health care workers was conducted at different hospitals in Saudi Arabia between April and May 2020. All the health workers who were able to participate in the study regardless of their COVID-19 infection status, including older adults, younger adults, and middle-aged adults were included in the study. The non-medical staffs were excluded in this study.

### Study Tool

The evaluation of the prevalence of FM among health worker was assessed by using the Fibromyalgia Rapid Screening Tool (FiRST) and London Fibromyalgia Epidemiological Study Screening Questionnaire (LFESSQ) questionnaires. The FiRST, a six-item questionnaire, was used to assessing the characteristics of pain, including location, character, associated symptoms, and quality of life effect. A cut-off score of 5 out of 6 is considered to be positive (corresponding to the number of positive items) (17). The LFESSQ, which consists of two parts, a pain component and a fatigue component, was another questionnaire (18). If they have a positive response to all four pain products and have either a positive response on the right and left side or a progressive outcome on either side, participants will satisfy the requirements for the pain portion. The questionnaire also asked about demographic features including sex, age, marital status, the sum of offspring, practice status, a comorbidities and vitamin D deficiency (defined as level of vitamin D <20 ng/ml).

### Statistical Analysis

The collected data was examined using the SPSS version 20 statistical software package (IBM SPSS). The data were entered into an excel file and transferred into SPSS. The results of the current study were then examined. The percentage analysis was conducted to determine the demographical statistics of the study participants. Descriptive statistics were used to summarize the data. Variables are conveyed as the mean ± standard deviation (mean ± SD). Clear-cut variables including sex, age cluster, marital status, the sum of offspring, practice status, specialty, comorbid, the status of healthcare service during the outbreak and FM were presented into frequencies and percentages. To effectively evaluate the proportions of the clear-cut variables between participants with and without fibromyalgia based on FiRST and LFESSQ criteria, the study employed the Chi-square test. Logistic regression exploration was conducted to identify predictors of fibromyalgia (FiRST score and LFESSQ criteria), where the prevalence of FM was taken as a binary dependent variable and independent variable as 11 covariates including demographic features and healthcare service during an outbreak. *P*-value <0.05 was identified as a statistically substantial outcome.

## Results

A total of 992 health workers completed the assessment visits for the physician's evaluation of FM. Prevalence of fibromyalgia during COVID-19 outbreak in Saudi Arabia is presented in Table 1.

**Table 1:** Prevalence of fibromyalgia during an outbreak (*n* = 992).

	***n* (%)**
**Prevalence of fibromyalgia (FiRST score)**	
Fibromyalgia (score ≥5)	125 (12.6)
No fibromyalgia (score <5)	867 (87.4)
**FiRST score distribution**	
6/6	58 (5.8)
5/6	67 (6.8)
4/6	114 (11.4)
3/6	109 (11.0)
2/6	101 (10.2)
1/6	79 (8.0)
0/6	464 (46.8)
**LFESSQ criteria**	
**Prevalence of fibromyalgia (LFESSQ)**	
Fibromyalgia (Pain alone or pain with fatigue)	196 (19.8)
No fibromyalgia	796 (80.2)
**LFESSQ criteria**	
Pain alone	196 (19.8)
Pain with fatigue	114 (11.5)
Fatigue alone	225 (22.7)
No pain no fatigue	685 (69.1)

*Values given in parentheses are percentages*.

*Out of 992 participants, using the FiRST criteria for the diagnosis of FM, 12.6% (n = 125) participants had a score of ≥5. On the other hand, using LFESSQ criteria, 19.8% (n = 196) of the participants had been diagnosed with fibromyalgia (pain alone or pain with fatigue) using the same cut-off*.

Table 2 presents the relationship between FM with demographic characteristics. A total of 992 healthcare professionals participated in the study, out of which 408 (41.1%) were males while 584 (58.9%) of those participants were females. The mean age was 37.3 years, and around 54 per cent (53.9%) of the participants were under 30–39 years age group. Three fourth of the participants were married (78.6%). The highest number of participants in this study was specialists (20.2%), followed by 16.6% of residents, while 15.2% were consultants. One-third of the participants (*n* = 319, 32.2%) have Vitamin D deficiency. Being a female (*p* < 0.001) with co-morbidities (*p* < 0.001) were the predictors of FM by using both the diagnostic criteria (LFESSQ & FiRST). Although most of the cases of FM were higher in the age group between 30 and 39 years, a significant association was seen in FM diagnosed on LFESSQ criteria (*p* = 0.044). No statistically significant differences were observed for age, marital status, practice status and a number of children.

**Table 2:** Relationship of fibromyalgia with demographic characteristics (*n* = 992).

**Characteristics**	**Total (%)**	**Fibromyalgia**			
	**(*n* = 992)**	**FiRST score** **(*n* = 125)**	***p*-value**	**LFESSQ (*n* = 196)**	***p*-value**
**Gender**					
Male	408 (41.1)	38 (30.4)	**0.009**	58 (29.6)	**0.000**
Female	584 (58.9)	87 (69.6)^*^		138 (70.4)^*^	
**Age (in years)**					
20–29	175 (17.6)	13 (10.4)	0.181	26 (13.3)	0.044
30–39	535 (53.9)	77 (61.6)		98 (50.0)^*^	
40–49	177 (17.8)	23 (18.4)		46 (23.4)	
50–59	85 (8.7)	9 (7.2)		20 (10.2)	
60 or above	20 (2.0)	3 (2.4)		6 (3.1)	
**Marital status**					
Single	176 (17.7)	21 (16.8)	0.313	41 (20.9)	0.408
Married	783 (78.9)	97 (77.6)		148 (75.5)	
Others	33 (3.4)	7 (5.6)		7 (3.6)	
**Number of children**					
None	285 (28.7)	29 (23.2)	0.106	51 (26.0)	0.269
One	707 (72.3)	96 (76.8)		145 (74.0)	
**Practice status**					
Consultant	151 (15.2)	20 (16.0)	0.416	33 (16.8)	0.287
Dentist	17 (1.7)	4 (3.2)		4 (2.0)	
Intern	19 (1.9)	3 (2.4)		5 (2.6)	
Lab worker	22 (2.2)	4 (3.2)		7 (3.6)	
Nurse	164 (16.5)	27 (21.6)		37 (18.9)	
Other	104 (10.5)	7 (5.6)		11 (5.6)	
Physician	119 (12.0)	16 (12.8)		27 (13.8)	
Physiotherapy	31 (3.1)	4 (3.2)		6 (3.1)	
Resident	165 (16.6)	19 (15.2)		31 (15.8)	
Specialist	200 (20.2)	21 (16.8)		35 (17.9)	
**Comorbidities**					
Yes	463 (46.7)	87 (69.6)^*^	**0.000**	128 (65.3)^*^	**0.000**
No	529 (53.3)	38 (26.2)		68 (34.7)	
**^∧^Previous history**					
Vitamin D def.	319 (32.2)	62 (49.6)	**0.000**	97 (49.5)	**0.000**
Diabetes mellitus	42 (4.2)	12 (9.6)	**0.000**	13 (6.6)	**0.000**
Hypothyroidism	36 (3.6)	9 (7.2)	**0.000**	10 (5.1)	**0.015**
Others	125 (12.6)	26 (20.8)	**0.000**	39 (19.9)	**0.000**

*Values given in parentheses are percentages*.

∧ *There may be more than on disease in a specific participant*.

* *Shows significant proportions at p ≤ 0.05; def, deficiency*.

**Figure 1:**
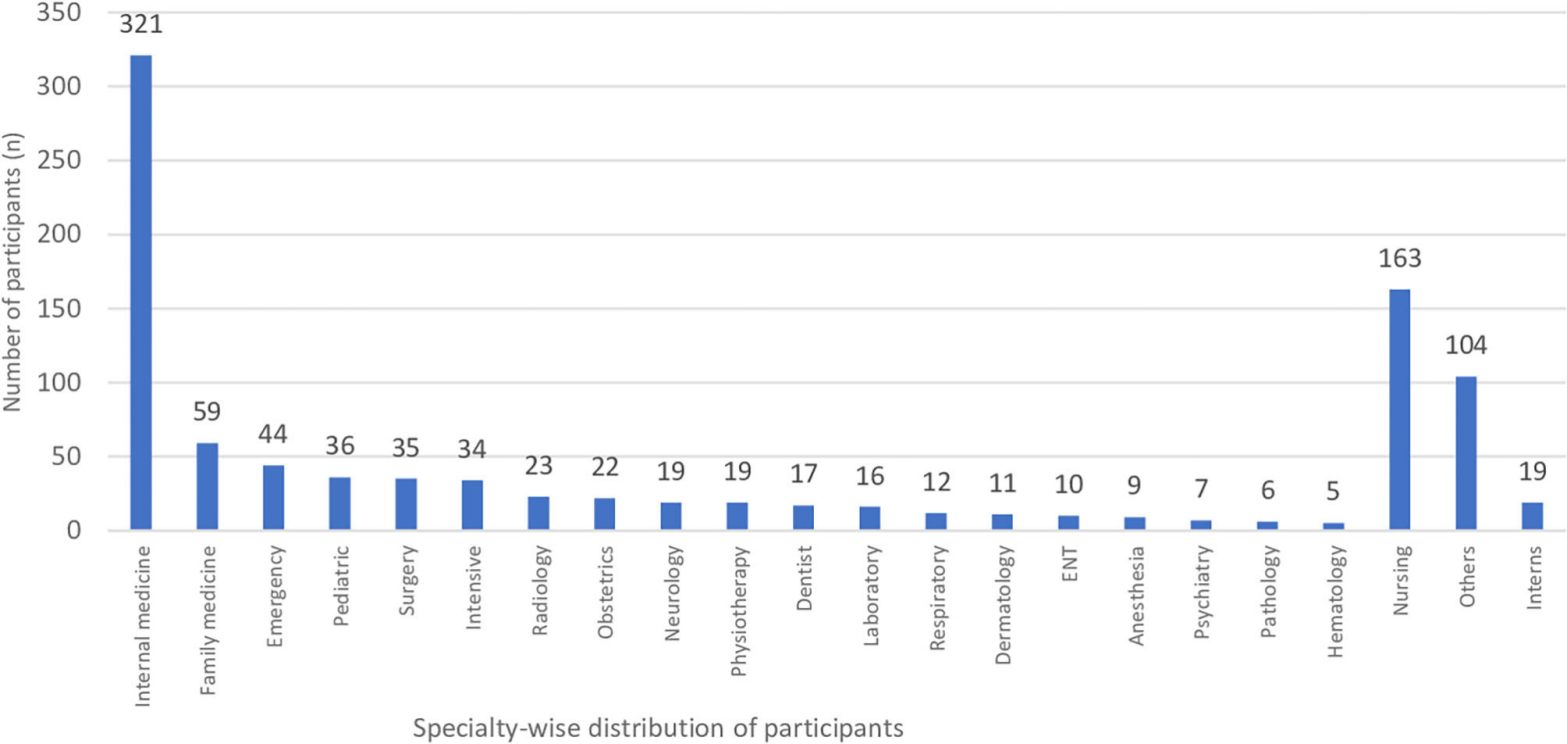
Specialty-wise distribution participants.

Relationship of FM with a specialty of the participants is presented in Table 3 and Figure 1. The commonest specialty of the participants was internal medicine (32.4%) followed by nursing (16.5%), and at least 5 (0.5%) participants belong to Hematology. An examination of the specialty of the participants and FM diagnosed using both FiRST (*p* = 0.358) and LFESSQ (*p* = 0.241) score determined minimal or no substantial statistical variances.

**Table 3:** Relationship of fibromyalgia with the specialty of the participants (*N* = 992).

**Specialty**	**Total (%)**	**Fibromyalgia**			
		**FiRST score** **(*n* = 125)**	***p*-value**	**LFESSQ (*n* = 196)**	***p*-value**
Internal medicine	321 (32.4)	33 (26.4)	0.358	55 (28.1)	0.241
Family medicine	59 (5.9)	7 (5.6)		13 (6.6)	
Emergency	44 (4.4)	4 (3.2)		7 (3.6)	
Pediatric	36 (3.6)	5 (4.0)		6 (3.1)	
Surgery	35 (3.5)	3 (2.4)		9 (4.6)	
Intensive	34 (3.4)	7 (5.6)		12 (6.1)	
Radiology	23 (2.3)	4 (3.2)		4 (2.0)	
Obstetrics	22 (2.2)	4 (3.2)		6 (3.1)	
Neurology	19 (1.9)	4 (3.2)		4 (2.0)	
Physiotherapy	19 (1.9)	3 (2.4)		5 (2.6)	
Dentist	17 (1.7)	4 (3.2)		4 (2.0)	
Laboratory	16 (1.6)	1 (0.8)		5 (2.6)	
Respiratory	12 (1.2)	1 (0.8)		1 (0.5)	
Dermatology	11 (1.1)	1 (0.8)		0 (0)	
ENT	10 (1.0)	2 (1.6)		3 (1.5)	
Anesthesia	9 (0.9)	1 (0.8)		2 (1.0)	
Psychiatry	7 (0.7)	1 (0.8)		3 (1.5)	
Pathology	6 (0.6)	3 (2.4)		2 (1.0)	
Hematology	5 (0.5)	0 (0)		2 (1.0)	
Nursing	163 (16.4)	27 (21.6)		37 (18.9)	
Others	104 (10.5)	7 (5.6)		11 (5.6)	
Interns	19 (1.9)	3 (2.4)		5 (2.6)	

*Values given in parentheses are percentages, Chi-square p-value*.

Table 4 reveals that out of 992 participants, 844 (85.1%) worked during the Covid-19 outbreak, 74.4% were in the hospital with suspected Covid-19 patients, 53.6% covered covid-19 patients, 48.8% covered in hospital on call, 60.5% were on call during daytime, 71% were in area quarantine. Using the FiRST Score cutoff, the prevalence of FM was significantly higher among those participants who worked during an outbreak (*p* = 0.043), when covering Covid-19 patient (*p* = 0.02), covered in-hospital on-call (*p* = 0.035), and while in quarantine (*p* = 0.035). On the other hand, using LFESSQ criteria, the prevalence of FM was higher among participants who worked in the daytime (*p* < 0.001), shifts (*p* = 0.034), and those who were quarantined (*p* = 0.003).

**Table 4:** Relationship of fibromyalgia with the healthcare service status of the participants during an outbreak (*n* = 992).

**Healthcare service**	**Total (%)**	**Fibromyalgia**			
	**(*n* = 992)**	**FiRST score** **(*n* = 125)**	***p*-value**	**LFESSQ (*n* = 196)**	***p*-value**
**Worked during an outbreak**					
Yes	844 (85.1)	114 (91.2)^*^	**0.043**	174 (88.8)	0.105
No	147 (15.1)	11 (8.8)		22 (11.2)	
**In hospital with Covid-19**					
Yes	738 (74.4)	100 (80.0)	0.125	150 (76.5)	0.444
No	254 (25.6)	25 (20.0)		46 (23.5)	
**Covered Covid-19 patient**					
Yes	532 (53.6)	79 (63.2)^*^	**0.022**	111 (56.6)	0.346
No	480 (46.4)	46 (36.8)		85 (43.4)	
**Covered in-hospital on call**					
Yes	484 (48.8)	72 (57.6)^*^	0.035	99 (50.5)	0.591
No	508 (51.2)	53 (42.4)		97 (49.5)	
**Work description**					
Home on-call	259 (26.1)	27 (21.6)	0.218	58 (29.6)	0.186
Daytime	600 (60.5)	76 (60.8)^*^	0.000	114 (58.2)^*^	0.000
Hospital on-call	166 (16.7)	25 (20.0)	0.730	29 (14.8)	0.374
Shift	283 (28.5)	40 (32.0)	0.130	58 (29.6)^*^	0.034
**Quarantine**					
Yes	704 (71.0)	99 (79.2)^*^	0.035	156 (79.6)^*^	0.003
No	288 (29.0)	26 (20.8)		40 (20.4)	

*Values given in parentheses are percentages*.

* *Shows significant proportions at p < 0.05*.

Table 5 reveals the predictors of FM associated with the healthcare service during Covid-19 pandemic. The analysis identified 12 predictors of fibromyalgia based on FiRST score criteria, whereas 7 predictors based on LFESSQ criteria. FiRST score reveals that females and age 31–39 years were about 2 times more likely to expose for FM. There was above 3 times more likely risk to suffer from FM among participants who had Vitamin D deficiency (*OR* = 3.12) and other diseases (*OR* = 3.39). Up to 2 times more likely to expose to FM those participants who worked during an outbreak, covered covid-19 suspected patients, covered in-hospital on call, shift-wise duty and presence in area quarantine, while up to 6 times more likely to suffer in daytime duty (*OR* = 5.91). LFESSQ criteria have shown almost likewise odd ratios of gender, age and presence of area quarantine, but it showed above 5.5 times more likely effect of vitamin D deficiency, other diseases, and up to 5 times more likely to suffer from FM if performed daytime duty.

**Table 5:** Predictors of fibromyalgia associated with the healthcare service during Covid-19 pandemic.

**Factors**	**Fibromyalgia** **FiRST score**		**Fibromyalgia** **LFESSQ**	
	**OR (95% C.I)**	***P*-value**	**OR (95% C.I)**	***P*-value**
Female gender	1.70 (1.14–2.55)^*^	**0.010**	1.87 (1.33–2.62)^*^	**0.000**
Age 31-39	1.61 (1.09–2.35)^*^	**0.016**	2.01 (1.18–3.44)	**0.010**
Vitamin D deficiency	3.12 (2.02–4.80)^*^	**0.000**	5.65 (3.76–8.48)^*^	**0.000**
Other disease	3.39 (1.97–5.84)^*^	**0.000**	5.86 (3.55–9.68)^*^	**0.000**
Worked during an outbreak	1.95 (1.02–3.71)^*^	**0.043**	1.49 (0.92–2.41)	0.105
In hospital with Covid-19	1.44 (0.90–2.28)	0.126	1.15 (0.80–1.66)	0.764
Covered Covid-19 patient	1.57 (1.07–2.31)^*^	**0.022**	1.16 (0.85–1.59)	0.347
Covered in-hospital on call	1.77 (1.21–2.58)^*^	**0.003**	1.09 (0.80–1.49)	0.591
Daytime duty	5.91 (3.69–9.46)^*^	**0.000**	4.87 (3.36–7.06)^*^	**0.000**
Shift duty	1.79 (1.10–2.91)^*^	**0.018**	1.47 (1.00–2.17)	0.051
Quarantined	1.65 (1.04–2.60)^*^	**0.031**	1.77 (1.21–2.58)^*^	**0.003**

* *Shows significant factors at p ≤ 0.05. OR (95% C.I): Odd ratio (95% confidence interval of odds ratios)*.

## Discussion

The pandemic of COVID-19 is a threat to international health. On the health care system, the COVID-19 pandemic places tremendous pressure with the ever-growing number of confirmed and suspected cases, hospital staffs who are at the frontline in the CoVID-19 outbreak, facing high risks of exposure to pathogens. Besides, the prolonged working hours of such continuous and heavy volume of work led to trigger fatigue, occupational burnout, psychological distress, and physical tiredness (22). These factors are significantly associated with the fibromyalgia. Fibromyalgia is related to a major decline in health-related quality of life (HRQOL) (23) and disability (24). Furthermore, it is critical to develop a strategy that consider all these factors in addition to availability of protective equipment, long working hours, rest, and psychological support (6, 25). Hence, the main focus of this study was to measure the prevalence of fibromyalgia during COVID outbreak among health workers in Saudi Arabia by using FiRST and LFESSQ criteria.

Our findings showed that the prevalence of fibromyalgia using FiRST and LFESSQ was 12.6 and 19.8%, respectively. Previously a study by Omair et al. (26) observed the prevalence of FM using the FiRST and LFESSQ was 6 and 11.6%, respectively, which is lower prevalence than the current study. However, our findings may not be comparable to the previous study due to methodological variations between the studies. Our study examined the prevalence of FM during CoVID-19 pandemic, and the population was representative of the different healthcare setting, and with higher sample size. On the other hand, the study by Omair et al. (26) evaluated the FM not during a pandemic, data collected in a single academic institution, while the sample size was small. These differences could probably demonstrate the variation in the prevalence between these studies.

In our study, the prevalence of fibromyalgia was higher in females when compared to males. This is because the woman was experiencing more distress, severe depression, and anxiety during the outbreak of COVID-19 (27). Prior studies demonstrated that the prevalence of fibromyalgia was higher in females (26, 28), which is similar to the present findings. A systematic review performed on various studies from around the world by Heidari et al. (29), in the general population, the pooled prevalence of FM among men and women was 0.01% (95% CI = −0.04, 0.06) and 3.98% (95% CI = 2.80, 5.20), respectively. This is probably due to the owing to their intimate, regular contact with patients and working longer hours than average, frontline nurses treating patients with COVID-19 were potentially at the highest risk of infection (27). In this study, most of the cases of FM were aged between 30 and 39 years, and these findings corroborate with previous studies that reported FM among middle-aged people (30, 31).

In current study, most of the respondents have Vitamin D deficiency, and our findings are in accordance to the study by Bhatty et al. (32) where vitamin D deficiency is frequently seen in patients diagnosed as FM. Our findings showed that the prevalence of fibromyalgia was highly associated with the specialty of the participants were internal medicine (32.4%) and nursing (16.5%). A study by Alnofaiey et al. (33) reported that the specialty of medical interns and microbiology/pathology/laboratory doctors had more difficulty in fall asleep during COVID-19, which is similar to present findings. In this study, the relationship of FM was significantly associated with the participants who worked during an outbreak, covered COVID-19 patient, covered in-hospital on call and in area quarantine. This is probably due to the SARS-CoV-2 could be acquired at work by health care workers through direct or indirect interaction with infected patients or other health care workers or as a result of ongoing transmission to the population (34). Finally, our study also identified the six common predictors of fibromyalgia associated with the healthcare service represented by both the questionnaires during COVID-19 pandemic, which included female gender, aged between 31 and 39 years, vitamin D deficiency, other diseases, daytime duty and area in quarantine. Similarly, the relationship among vitamin D levels, female, age with the fibromyalgia were determined by several studies (35–37).

Our study had some limitations. The cross-sectional study design makes it difficult to recognize the fibromyalgia risk. We have not confirmed fibromyalgia diagnosis using gold standard (physician assessment). Finally, it is possible to select a bias and overestimates the questionnaire responders. Despite these limitations, we think that our study adds great value to the limited literature on FM among healthcare workers, by being the first to measure the prevalence of FM among health workers in Saudi Arabia during COVID-19 pandemic and identify its important correlates.

## Conclusions

Patients with fibromyalgia continued to report a high level of disease burden, especially during COVID-19 outbreak. In this study, we have presented data on the prevalence of fibromyalgia during COVID outbreak among healthcare workers in Saudi Arabia. These findings have possible importance that may support the prevention of fibromyalgia among healthcare workers during the COVID-19 pandemic in order to maintain health. However, as COVID-19 pandemic continues, more future studies with larger population size need to be confirmed and investigated.

## Data Availability Statement

The raw data supporting the conclusions of this article will be made available by the authors, without undue reservation.

## Ethics Statement

The studies involving human participants were reviewed and approved by the study was conducted according to the guidelines of the Declaration of Helsinki and approved by Scientific Research Ethics Committee at Qatif Central Hospital (QCH-SREC0198/2020). The patients/participants provided their written informed consent to participate in this study.

## Author Contributions

SA and GA: conceptualization. SA and MA: data curation. AA: formal analysis. IS: methodology and software. SA: project administration. AA and GA: supervision. FA: validation and writing—original draft. GA: writing—review and editing. All authors contributed to the article and approved the submitted version.

## Funding

This research was funded by the Deanship of Scientific Research at Princess Nourah Bint Abdulrahman University through the Fast-track Research Funding Program.

## Conflict of Interest

The authors declare that the research was conducted in the absence of any commercial or financial relationships that could be construed as a potential conflict of interest.

## Publisher's Note

All claims expressed in this article are solely those of the authors and do not necessarily represent those of their affiliated organizations, or those of the publisher, the editors and the reviewers. Any product that may be evaluated in this article, or claim that may be made by its manufacturer, is not guaranteed or endorsed by the publisher.
